# Global prevalence and incidence of hallux valgus: a systematic review and meta-analysis

**DOI:** 10.1186/s13047-023-00661-9

**Published:** 2023-09-20

**Authors:** Yangting Cai, Yuke Song, Mincong He, Wei He, Xianxin Zhong, Hao Wen, Qiushi Wei

**Affiliations:** 1 Guangzhou University of Chinese Medicine, NO.12, Jichang Road, Baiyun District, Guangzhou, 510405 P. R. China; 2 The Third Affiliated Hospital of Guangzhou University of Chinese Medicine, NO.261, Longxi Road, Liwan District, Guangzhou, 510378 P. R. China; 3 Guangdong Research Institute for Orthopedics & Traumatology of Chinese Medicine, NO.261, Longxi Road, Liwan District, Guangzhou, 510378 P. R. China

**Keywords:** Hallux valgus, Prevalence, Incidence, Systematic review, Meta-analysis

## Abstract

**Background:**

Though hallux valgus is a common foot deformity, the integrated information on its global prevalence and incidence is relatively lacking. The aim of this research was to assess the global prevalence and incidence of hallux valgus, thus providing reliable data reference for clinical practice.

**Methods:**

A systematic review of global hallux valgus research publications concerning its prevalence and incidence was performed based on six electronic databases ((PubMed, Embase, Cochrane Library, Chinese National Knowledge Infrastructure (CNKI), China Online Journals and CQVIP)) from their inception to November 16, 2022. The search terms included “hallux valgus or bunion and prevalence or incidence or epidemiology.” All languages were included. Data were extracted by country, continent, age group, gender and other information. The risk of bias was assessed by the Joanna Briggs Institute Critical Appraisal Instrument for Studies Reporting Prevalence Data by using random-effects models to synthesize available evidence.

**Results:**

A total of 45 studies were included in the meta-analysis. The overall pooled estimated prevalence was 19% (95% CI, 13% to 25%) (*n=*186,262,669) for hallux valgus. In subgroup meta-analyses, the prevalence of hallux valgus was 21.96% (95% CI, 10.95% to 35.46%) in Asia, 3% (95% CI, 0% to 15%) in Africa, 18.35% (95% CI, 11.65% to 26.16%) in Europe, 29.26% (95% CI, 4.8% to 63.26%) in Oceania, and 16.1% (95% CI, 5.9% to 30.05%) in North America, respectively. The pooled prevalence of hallux valgus by gender was 23.74% (95% CI, 16.21% to 32.21%) for females and 11.43% (95% CI, 6.18% to 18%) for males. The prevalence was 11% (95% CI, 2% to 26%) in individuals younger than 20 years old, 12.22% in adults aged 20-60 years (95% CI, 5.86% to 20.46%) and 22.7% in elderly people aged over 60 years (95% CI, 13.1% to 33.98%).

**Conclusion:**

This research provided the global prevalence and incidence of hallux valgus in terms of its spatial, temporal, and population distribution. The global estimated pooled prevalence and incidence of hallux valgus was 19%. A higher prevalence of hallux valgus was found in females, Oceania countries, and among people aged over 60 years. Due to the high heterogeneity of the included studies, the findings should be interpreted with caution.

**Supplementary Information:**

The online version contains supplementary material available at 10.1186/s13047-023-00661-9.

## Background

Hallux valgus (HV) is a common foot deformity that causes bunions and difficulty walking in footwear [1]. It affects individuals of all ages [2]. In recent years, more attention has been paid to HV research, but primarily focuses on the pathogenesis and clinical treatment of the condition [3]. Osteophytes and thickening of the articular capsule are the main pathological changes in HV. There are more than 100 operative procedures for HV deformity [4], and every technique with its own advantages has solved some clinical problems.

As is known to us all, integrated information about the global prevalence of HV is relatively lacking. A previous systematic review and meta-analysis reported the prevalence of HV in 2010 [5], nevertheless, Nix [5] only searched 3 electronic databases (Medline, Embase, and CINAHL) and did not search any Chinese databases. In fact, as a country with the largest population, a lot of people in China are suffering from HV [6]. Globally, the research on overall prevalence of HV by classification and region of origin is still lacking. What is more, there is an increasing large number of new publications on epidemiologic evidence in some other counties and new regions in the last 12 years. To fill this gap of knowledge, a systematic review and meta-analysis of study was performed to report the prevalence and incidence of HV at the global level and to analyze the factors potentially between prevalence and possible related variables (e.g., gender, geographic location and age distribution) in terms of its spatial, temporal, and population distribution.

## Methods

### Literature search

The literature search was performed by the first author of this paper by using six electronic databases (PubMed, Embase, Cochrane Library, CNKI, China Online Journals and CQVIP) from their inception to November 16, 2022. The searches of this systematic review and meta-analysis were conducted according to Preferred Reporting Items for Systematic Reviews and Meta-Analyses (PRISMA) reporting guidelines [7, 8]. The search terms included “hallux valgus or bunion and prevalence or incidence or epidemiology” (the search strategy was provided in eAppendix 1 in the Supplementary File). All languages were included.

### Inclusion and exclusion criteria

The inclusion criteria were formulated as follows: (1) original research articles worldwide; (2) all studies with a cross-sectional, prospective cohort study, case-control, or cohort design; and (3) only studies that reported or sufficient data to calculate the prevalence of HV were included. There were no restrictions regarding languages. If there was more than one study among the same population, only the largest and latest one was brought into the review. Studies were excluded if they had (1) no relation to HV, foot deformities, or prevalence; (2) descriptions of operative or non-operative interventions; (3) studies related to specific disease groups (Rheumatoid Arthritis, diabetes, thyroid, and neuromuscular disorders), discussions of traumatic injury to the first toe joint; (4) they were review articles, conference abstracts, short communications, case reports, clinical opinions, letters, posters, or laboratory researches; (5) they did not report sufficient data, and efforts to contact the authors were unsuccessful; or (6) no access to obtain full text.

### Data extraction and quality assessment

Titles and abstracts were screened by two review authors (Y.T.C and Y.K.S). If the study was of relevance, it was selected for full-text review. Reviewers collected data from the full text independently and cross-checked the data. This was to ensure that the correct article was identified for full-text screening. Queries were discussed and resolved by a third review author (H.W.) if there was a disagreement. The following collected data included authors, the year of publication, countries or area in which countries were studied, continents, sample size, gender and age range, year of investigation, diagnosis, the number of participants affected by HV, and prevalence of HV. The authors were contacted if more information was required or unclear .

The risk of bias of the included studies was independently assessed by using the Joanna Briggs Institute Critical Appraisal Instrument for Studies Reporting Prevalence Data (JBI) [8] by two of the authors (Y.T.C and Y.K.S), which was proven to be a reliable and valid tool for assessing observational studies. Risk of bias was categorized as high when the study reaches up to 49% score “yes”, moderate when the study reached 50% to 69% score “yes”, and low when the study reached more than 70% score “yes” (eTable 1 in the Supplement).

### Data analysis

The metaprop module in the R statistical software package, version 4.2.2, was used for the summary statistic. The logit method was chosen to transform each prevalence proportion of study or subgroup with HV, then adopted an inverse-variance-weighted random-effects meta-analysis. HV prevalence (with 95% CIs) for the overall population and subgroups were calculated. The results were visualized using forest plots. The *I*^2^ statistic for heterogeneity and the *P* value for heterogeneity (Cochrane Q statistic) were showed in forest plots. A funnel plot was used to assess publication bias by using the Egger test. Since the study populations were diverse, prevalence estimates were only pooled between studies with similar continents, gender and age characteristics. The prevalence of HV among the overall population and among subgroups were reported.

## Results

### Literature search and included studies

**Fig. 1:**
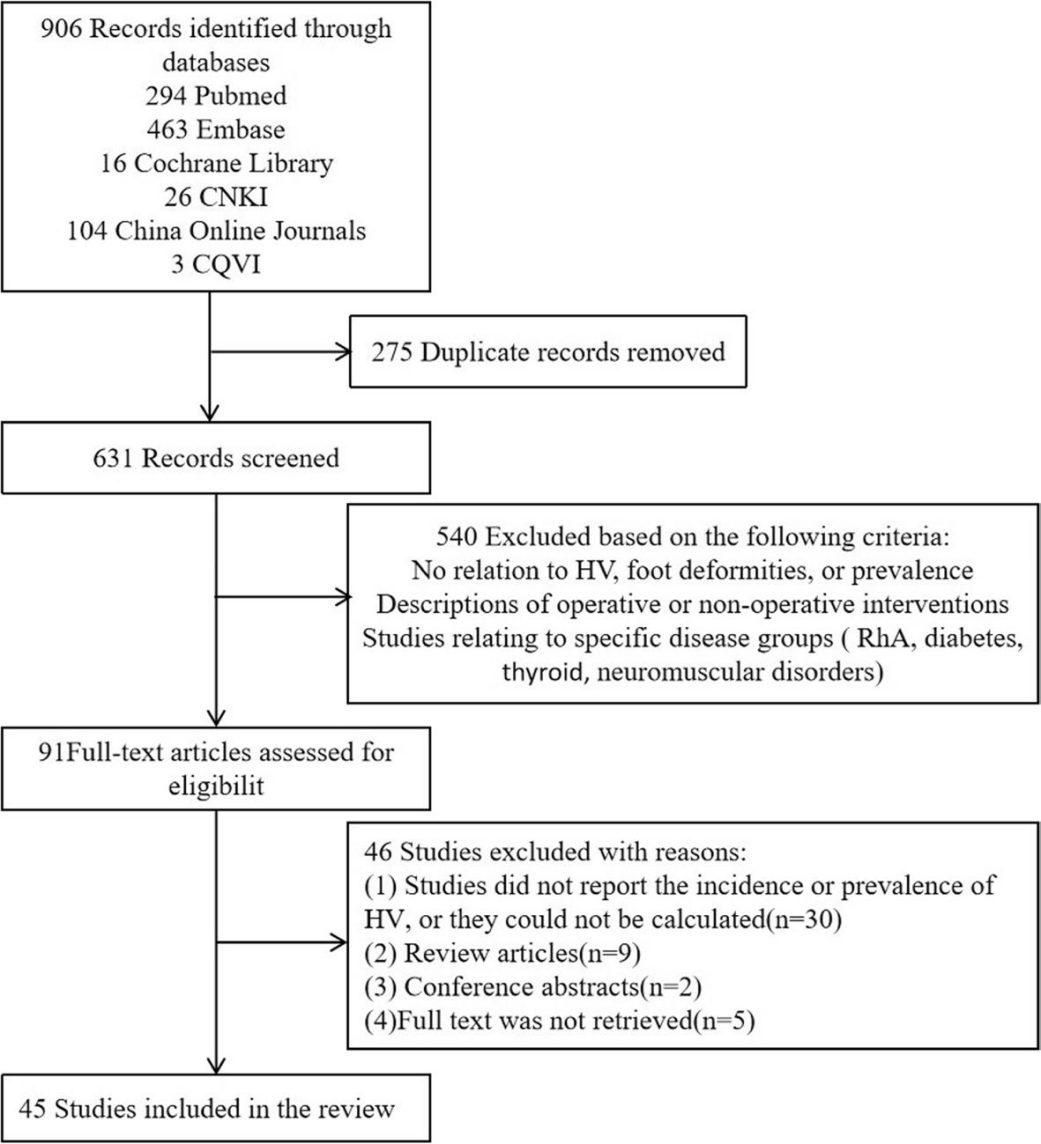
PRISMA flow diagram. HV: hallux valgus; PRISMA: Preferred Reporting Items for Systematic Reviews and Meta-analyses; RhA: Rheumatoid Arthritis; CNKI: https://www.cnki.net/; China Online Journals: https://www.wanfangdata.com.cn/; CQVIP: http://www.cqvip.com/

The search of the databases resulted in 906 records from the six databases. Search history was shown in the PRISMA flow diagram (Fig. 1). A total of 44 papers [9–52] were included (*n=*186,262,669). These epidemiological studies came from five continents and 17 different national regions (Table 1). There was a wide variation in study characteristics in terms of the population studied and the methodology employed. Three studies were conducted in Africa, 15 studies in Asia, 18 studies in Europe, five studies in North America and four studies in Oceania. Among these 45 studies, nine reported the prevalence of HV in China, one in France, one in Germany, three in Nigeria, one in Indonesia, three in Japan, one in Korea, one in Netherlands, one in New Guinea, one in Greece, one in Poland, four in Spain, one in Turkey, nine in UK, five in USA, and three reported the prevalence of HV in Australia.

**Table 1:** Characteristics of 45 studies of hallux valgus

Authors	Year	Country	Continent	No.		Prevalence(%)	Age	Study period	Diagnosis
				HV	Total				
XU XJ [9]	2021	China	Asia	1	143	0.70%	30-39	2015-2018	Q
Wu HT [10]	2007	China	Asia	98 M:5 F:93	1233 M:387 F:846	7.95%	18~74	NR	C
Sayli U [11]	2018	Turkey	Asia	1443 F:951 M:492	2662 F:1615 M:1047	54.30%	18-96	2016	Q+X
Akinobu N [12]	2013	Japan	Asia	120 M:21 F99	403 M:135, F:268	29.80%	65–94	2012	Q
Nathaniel G [13]	1980	USA	North America	23697509	186000000	12.74%	All ages	1978-1979	Q
Cui B [14]	2012	China	Asia	79 M:10 F:69	779 M: 311 F468	10.14%	≥16	2012	X
Dunn JE [15]	2004	USA	North America	291	784 M:339, F:445	37.1%	65-101	2001-2002	C
Maclennan R [16]	1966	New Guinea	Oceania	42 M:10 F:32	1256 M:665 F:591	3.34%	≥10	1964	C
Hylton B [17]	2005	Australia	Oceania	38 M:8 F:30	176 M:56 F:120	21.59%	62-96	NR	Q+C
Pita Fernandez S [18]	2014	Spain	Europe	149 M:54 F:95	1002	38.00%	≥ 40	2009-2012	Q+C
David W [19]	2014	Greece	Europe	113	2096 M:1272 F:824	5.40%	9.9 - 65.1	2011	C
David W [19]	2014	USA	North America	937	7192 M:4279 F:2913	13.00%	8.0-84.0	2007-2011	C
Gonzalez-Martin C [20]	2017	Spain	Europe	703 M:233 F:470	1837 M:840 F997	39.00%	≥40	2009-2012	C+Q
Scott G [21]	2007	Australia	Oceania	31	100	31.00%	All ages	2006	C+X
Hong K [22]	2003	China	Asia	27	33	81.82%	14.13-15.2	1993	Q
Matsumoto T [23]	2022	Japan	Asia	766 M:149 F:617	1996 M:654 F:1342	38.38%	All ages	2018-2019	X+S
Cobos-Moreno P [24]	2022	Spain	Europe	21	53 M:32 F: 21	40.00%	average age: 27.5	2018-2019	C+Q
Menz HB [25]	2021	UK	Europe	450	1482 F:739 M:743	30.40%	≥50	2021	C
Dittmar JM [26]	2021	UK	Europe	31 M:20 F11	177	18.00%	NR	NR	C+X
Bafor A [27]	2020	Nigeria	Africa	11 M:7 F:4	1758 M:814 F:944	0.60%	5-13	in medieval	C
Yuan YF [28]	2020	China	Asia	359 M: 103 F: 256	1193 M:494 F:699	30.10%	≥60	2019	Q
Puszczalowska-Lizis E [29]	2019	Poland	Europe	109	300 All female	36.33%	30–40	2019	C
Montiel V [30]	2017	Spain	Europe	85 F:72 M:13	254 M:86 F:168	33.50%	2–81	2007-2009	C+X
Soemarko DS [31]	2019	Indonesia	Asia	35	191 all female	18.32%	All age	NR	C+X
Hendry GJ [32]	2018	UK	Europe	75 F:72 M:3	593 F:399, M:192	12.60%	31-52	2018	Q+S
Huang ZG [33]	2006	China	Asia	22 M:9 F:13	319 M:156, F:163	6.90%	17.5-22.3	2000	C
Dufour AB [34]	2017	USA	North America	1242 M:323 F:919	4884 M:215 F: 2738	25.40%	≥60	2002–2008	C
Mumido [35]	2014	China	Asia	118 M:17 F:101	2014 M:909, F:1105	5.90%	16-83	2012	C
Zhang YZ [36]	2004	China	Asia	93	1033	9.00%	15-45	NR	C+X
hine LB [37]	1965	UK	Europe	444	3515 M:1852 F:1663	12.63%	≥5	1905	C+X
Roddy E [38]	2008	UK	Europe	1194 F:853 M:34	4249 F:2445, M:1804	28.4%	30-95	NR	C+X
Spahn G [39]	2004	Germany	Europe	83	2368	3.50%	13-18	NR	S
Menz HB [40]	2015	UK	Europe	163	517 F:287 M:230	31.53%	≥50	NR	C+X
Menz HB [41]	2010	UK	Europe	49	4500	1.10%	All ages	2006	C+X
Cho NH [42]	2009	Korea	Asia	364 F:221 M:143)	563 M:245 F:318	64.70%	40-69	2007	X
Mafart B [43]	2007	France	Europe	132	605	21.80%	≥30	5-17 century	X+Q
Jerosch J [44]	1998	UK	Europe	59	345	17.10%	10-13	NR	C
kuda H [45]	2014	Japan	Asia	102	343 all female	29.7%	≥20	2010-2012	Q+X
Menz HB [46]	2001	Australia	Oceania	100	135 M:55 F:80	74.00%	75-93	NR	Q+X
Krul M [47]	2009	Netherlands	Europe	23 M:4 F:19	87952	0.03%	0–17	2001	C
Menz HB [48]	2011	UK	Europe	974 F:696 M:278	2681	36.3%	>56	NR	C
Enwemeka CS [49]	1984	Nigeria	Africa	20 M:6 F:14	3144 M:1444, F1700	6.36%	2.75-30.50	NR	C
Chou LW [50]	2008	China	Asia	252 F:206 M:46	18,006 M:8883 F:9123	1.40%	6-12	2006	C
Owoeye BA [51]	2021	Nigeria	Africa	149 F:84 M:65	970 M:499 F:471	15.40%	11-40	NR	Q+X
Shibuya N [52]	2011	USA	North America	2130	96833 M:45610 F:51223	2.20%	>10	1990	Q+X

*M* Mal, *F* Female, *UK* United Kingdom, *USA* United States, *Q* Questionnaire, *C* Clinical examination, *X* X-ray examination, *NR* not reported

### Overall prevalence of HV

**Fig. 2:**
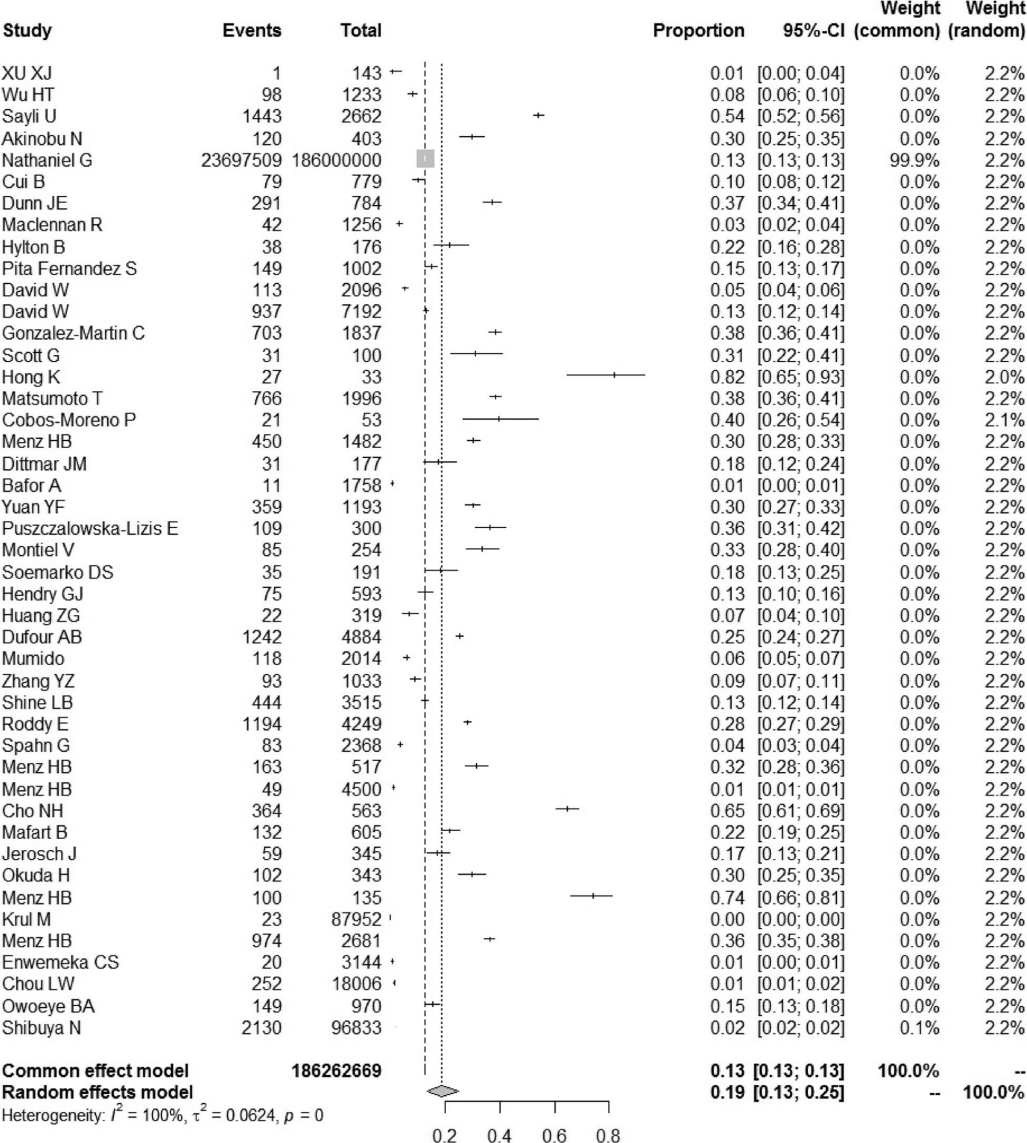
Forest plot of the overall prevalence of hallux valgus. Confidence intervals and pooled prevalence estimate for HV. The analysis was conducted using a random-effects model

The pooled prevalence estimates for HV were shown in Fig. 2. The studies reported prevalence and incidence of HV ranged from 1% to 82%. The random-effects overall pooled estimated prevalence was 19% (95% CI, 13% to 25%). The heterogeneity was very high (*I*^2^=100%; *P*=0). It was found that no study disproportionately affected the overall result. According to the Egger test (*P*=0.45), there was no publication bias existed.

### Prevalence of HV by year of publication

**Fig. 3:**
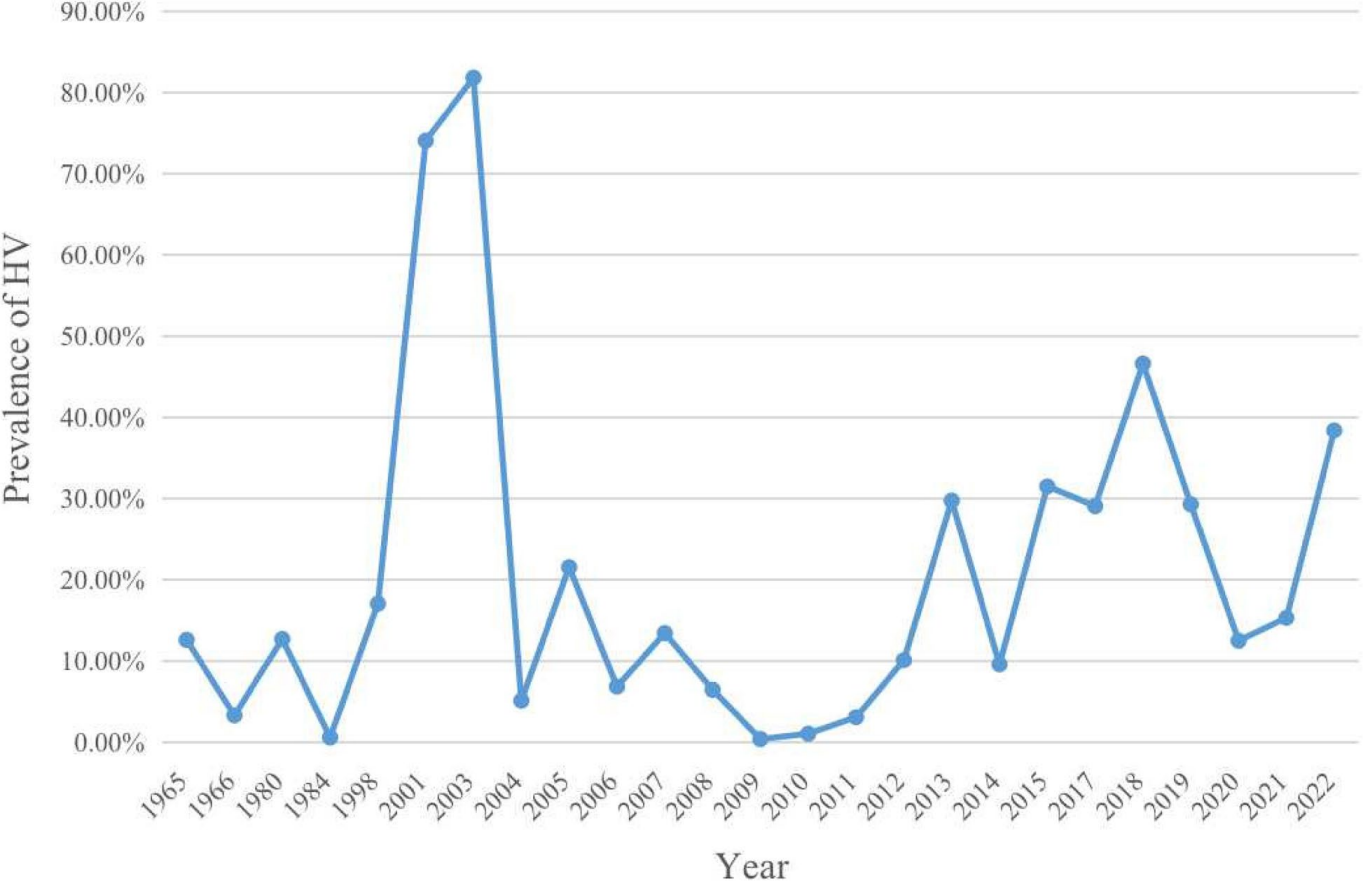
Relationship between prevalence of HV and publication tear. The pooled prevalence estimates from random effects models for studies providing data for specific year groups. Each point represents the pooled prevalence estimate for that year group

To study the relationship between the year of publication and the prevalence of HV, an association analysis on publication year was conducted. The results indicated that the prevalence of HV was different in publication year from Fig. 3, ranging from 81.82% in 2003 to 0.03% in 2009. According to the figure, there was no consistent trend apparent and no statistically significant relationship between HV prevalence and year of publication.

### Prevalence of HV by continent

**Fig. 4:**
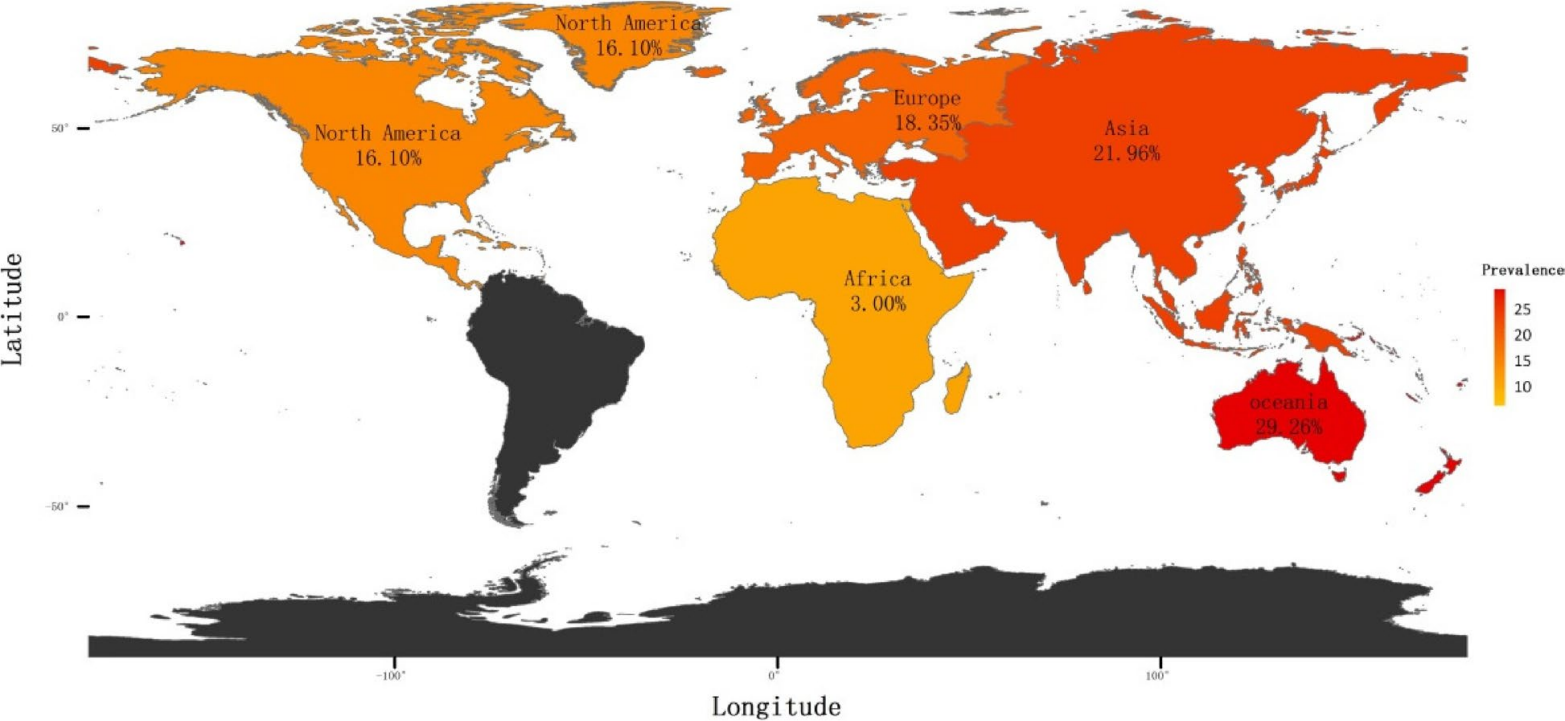
The distribution world map of the prevalence of HV. The world map displays the distribution of the prevalence of HV in different regions of the world. The darker shades indicate higher prevalence rates

In the comprehensive review of the studies, a global distribution was observed. A significant proportion of the epidemiological investigations originated from Europe, encompassing seven countries (41.18%) and 18 research articles (38.93%), with a collective sample size of 114,526. In contrast, Africa was represented by a mere three studies (6.67%), involving 5,872 participants. Meanwhile, Asia contributed 15 investigations (33.33%) with a total of 30,911 subjects, and North America was the source of five studies (11.11%) with an extensive sample population of 186,109,693. Lastly, Oceania was represented by four studies (8.89%), encompassing 1,667 participants. The largest sample of epidemiological survey was in the United States from 1978 to 1979. The prevalence of HV was 3% (95% CI, 0% to 15%) in Africa (with very high heterogeneity [*I*^2^=99%; *P*<0.01]), 21.96% (95% CI, 10.95% to 35.46%) in Asia (with very high heterogeneity [*I*^2^ =100%; *P*=0]), 18.35% (95% CI, 11.65% to 26.16%) in Europe (with very high heterogeneity [*I*^2^=100%; *P*=0]), 16.1% (95% CI, 5.9% to 30.05%) in North America (with very high heterogeneity [*I*^2^=100%; *P*=0]) and 29.26% (95% CI, 4.8% to 63.26%) in Oceania (with very high heterogeneity [*I*^2^=99%; *P*<0.01]) (eFigures 1-5 in the Supplement). The global prevalence and incidence of HV was illustrated in Fig. 4 in various areas of the world from the geographical distribution map.

### Prevalence of HV by gender

Meta-analysis by gender in the subgroups consistently showed gender differences in the prevalence of HV. Only 24 studies reported the number of men and women with and without HV. Only two studies were all females. There was a total of 28,330 females (55.62%) and 22,608 males (44.38%). The pooled prevalence of HV by gender was 23.74% (95% CI, 16.21% to 32.21%) for females (with very high heterogeneity [*I*^2^=100%; *P*=0]) and 11.43% (95% CI, 6.18% to 18%) for males (with very high heterogeneity [*I*^2^=99%; *P*=0]) (eFigures 6, 7 in the Supplement).

### Prevalence of HV by age

The results of studies were grouped by age of study population. 10 studies reported individuals younger than 20 years old, seven studies included individuals 20 to 60 years old, and eight studies among aged over 60 years old. The prevalence was 11% (95% CI, 2% to 26%) in individuals younger than 20 years old (with very high heterogeneity [*I*^2^=99%; *P*<0.01]), 12.22% (95% CI, 5.86% to 20.46%) in adults aged 20 to 60 years old (with very high heterogeneity [*I*^2^=97%; *P*<0.01]) and 22.7% (95% CI, 13.1% to 33.98%) in elderly people aged over 60 years old (with very high heterogeneity [*I*^2^=100%; *P*=0]) (eFigures 8-10 in the Supplement).

### Quality assessment

Overall, the quality of the studies included in this systematic review was moderate to high. The majority of studies reached more than 70% score “yes”, indicating that they were of high methodological quality. However, some studies had limitations, such as small sample sizes or inadequate reporting of results, which may have affected the validity of their findings. To ensure the robustness of this meta-analysis, sensitivity analyses were conducted to exclude studies that scored low on the JBI quality assessment. A funnel plot was used to assess publication bias using the Egger test. According to the Egger test (*P*=0.45), it showed that there was no statistically significant publication bias (*P*>0.05) (eFigure 11 in the Supplement). These sensitivity analyses did not materially affect the overall results, indicating that the findings of this meta-analysis were robust to variations in the quality of the included studies. In conclusion, the quality of the studies included in this systematic review and meta-analysis was generally good, with most studies meeting the majority of the JBI quality assessment criteria.

## Discussion

This research revealed the global prevalence and incidence estimates of HV. The results described that the global estimated pooled prevalence and incidence of HV was 19%. However, there were differences among all subgroups in this meta-analysis. The prevalence of HV was higher in females compared with males, as well as increasing trends in age, the incidence rate of HV was more prevalent in elderly people aged over 60 years old. The prevalence of HV varied greatly around the world, with high prevalence in Asia and Oceania. According to the present study, there was no significant relationship between prevalence of HV and publication year. The results should be carefully taken into account for the high heterogeneity of the included studies.

This meta-analysis revealed a high heterogeneity in HV prevalence estimates. Several possible reasons were related to a number of factors, such as diagnosis of HV, different regions and races of study location, gender, age and study quality [5]. Some investigators had no knowledge of diagnostic criteria, for the clinical diagnosis of HV resulting from the variety of clinical and radiographic features, thus it was important for the HV diagnosis to depend on physician experience. In fact, the methods of HV diagnosis may be main reasons of the substantial differences. On top of that, methodological heterogeneity may be another factor which was important to take into account. This data of prevalence and incidence came from different study designs or methodological quality. The heterogeneity of studies included sampling methods, sample sizes and data collection.

To the best of our knowledge, this study was the first systematic review and meta-analysis to show the world map on the distribution of the prevalence of HV. This set of epidemiologic studies was obtained from a variety of global locations, including the continents of Africa, Asia, Europe, North America, and Oceania, providing a comprehensive worldwide survey. In continent subgroup analysis, comparative analysis revealed a higher prevalence of HV in countries within the Asia and Oceania regions, as opposed to those observed globally. A study from China reported the highest prevalence of HV (81.82%) among 272 gymnasts [22], while the lowest prevalence (0.03%) was reported in a sample of 273 adults from the Netherlands who were not specifically selected for their involvement in sport [47]. Certain exercise patterns that are prevalent among athletes may contribute to the development of HV, although further research is needed to establish a causal relationship. The socioeconomic and sociocultural differences might be factors among regions of study location. Different continents and countries may have different sampling methods. It has been reported that variations in the prevalence of HV are also related to a number of factors, including regional variation, ethnicity and shoe wearing [53].

Subgroup analysis by gender confirmed that the prevalence of HV was higher among females than among males, with a pooled estimate of 23.74% compared to 11.43%. Women who wear high-heeled shoes exhibit higher plantar pressure on the hallux compared to men who wear flat shoes [54, 55]. This factor may explain why the higher prevalence of HV was found in females than males.

Subgroup analysis by age showed that the prevalence of HV was highest among the elderly people aged over 60 years. The results of the present study clearly showed an increase in the prevalence of HV with age: 11% in individuals younger than 20 years old, 12.22% in adults aged 20 to 60 years and 22.7% in elderly people aged over 60 years. This trend could be associated with life-style and age-related changes (e.g., ligament laxity, a greater reduction in size of skeletal muscle, loss of muscle strength, abductor hallucis muscle size and quality) [56, 57].

## Limitations

Although the strengths of this study include study selection, a double review process, data extraction, stringent selection criteria, and the assessment of quality by two independent reviewers, there are still several limitations in this study. First, random error is hard to avoid, although the sample size of this study was one of the largest collected currently (*n=*186262669). Certain databases that could provide additional epidemiological data (e.g., grey literature) were not included. Furthermore, numerous studies lacked granular features such as age stratification, ethnicity, geographic location, and sampling methodology. Third, corresponding authors of some articles could not be reached. Finally, this systematic review study had a high degree of heterogeneity. Additionally, studies without full-texts available were removed. Those might affect the pooled estimates of prevalence.

## Conclusions

This research provided the global prevalence and incidence of HV in terms of its spatial, temporal, and population distribution. It showed that the global estimated pooled prevalence of HV was 19%. A higher prevalence of HV was found in females (23.74%). The findings in this study also confirmed that HV was more prevalent in the elderly. The global prevalence and incidence of HV was found higher in Oceania countries from the geographical distribution world map. Due to the high heterogeneity of the included studies, the findings should be interpreted with caution.

### Supplementary Information

**Additional file 1:** **eFigure 1.** Forest Plot of the Prevalence of HV by Afria. **eFigure 2.** Forest Plot of the Prevalence of HV by Asia. **eFigure 3.** Forest Plot of the Prevalence of HV by Europe. **eFigure 4.** Forest Plot of the Prevalence of HV by North America. **eFigure 5.** Forest Plot of the Prevalence of HV by Oceania. **eFigure 6.** Forest Plot of the Prevalence of HV by male. **eFigure 7.** Forest Plot of the Prevalence of HV by female. **eFigure 8.** Forest Plot of the Prevalence of HV by 0-20years. **eFigure 9.** Forest Plot of the Prevalence of HV by 21-60year. **eFigure 10.** Forest Plot of the Prevalence of HV by 61 year older. **eFigure 11.** Egger test. **eTable 1.** Quality assessment. **Appendix 1.** Search Strategy.

## Data Availability

The original contributions presented in the study were included in the article/Supplementary Material, further inquiries can be directed to the corresponding author/s.

## Abbreviations

HV: Hallux valgus
PRISMA: Preferred Reporting Items for Systematic Reviews and Meta-Analyses
JBI: Joanna Briggs Institute
RhA: Rheumatoid Arthritis
M: Male
F: Female
UK: United Kingdom
USA: United States
Q: Questionnaire
C: Clinical examination
X: X-ray examination
NR: Not reported
