# Supplementary material for: Global prevalence and incidence of hallux valgus: a systematic review and meta-analysis
Source: J Foot Ankle Res. 2023 Sep 20;16:63. doi: 10.1186/s13047-023-00661-9 (PMC10510234; doi:10.1186/s13047-023-00661-9)
Supplement: Supplementary file 1 — Additional file 1: eFigure 1. Forest Plot of the Prevalence of HV by Afria. eFigure 2. Forest Plot of the Prevalence of HV by Asia. eFigure 3. Forest Plot of the Prevalence of HV by Europe. eFigure 4. Forest Plot of the Prevalence of HV by North America. eFigure 5. Forest Plot of the Prevalence of HV by Oceania. eFigure 6. Forest Plot of the Prevalence of HV by male. eFigure 7. Forest Plot of the Prevalence of HV by female. eFigure 8. Forest Plot of the Prevalence of HV by 0-20years. eFigure 9. Forest Plot of the Prevalence of HV by 21-60year. eFigure 10. Forest Plot of the Prevalence of HV by 61 year older. eFigure 11. Egger test. eTable 1. Quality assessment. Appendix 1. Search Strategy. [file 13047_2023_661_MOESM1_ESM.zip › 13047_661_Supplementary Materials and Appendix/Appendix 1 Search Strategy.docx]

Global Prevalence and Incidence of Hallux Valgus: A Systematic Review and Meta-analysis

**Appendix 1: Search Strategy**

**PUBMED:**

((((Hallux Abductovalgus[Title/Abstract]) OR (Hallux Valgus[Title/Abstract])) OR (Bunion[Title/Abstract])) OR (Bunions[Title/Abstract])) AND (((Incidence[Title/Abstract]) OR (Prevalence[Title/Abstract])) OR (Epidemiology[Title/Abstract]))

10 Nov 2022

**Embase**

No. Query Results Results Date

1. 'hallux valgus'/exp OR 'hallux valgus' OR 'hallux 6,420 11 Nov 2022

abductovalgus':ab,ti OR bunion:ab,ti OR

bunions:ab,ti

2. 'incidence':ab,ti OR 'prevalence':ab,ti OR 2,372,585 11 Nov 2022

'epidemiology':ab,ti

1. 1 AND 2 463 11 Nov 2022
