# Supplementary figures and images for: Global prevalence and incidence of hallux valgus: a systematic review and meta-analysis
Source: J Foot Ankle Res. 2023 Sep 20;16:63. doi: 10.1186/s13047-023-00661-9 (PMC10510234; doi:10.1186/s13047-023-00661-9)

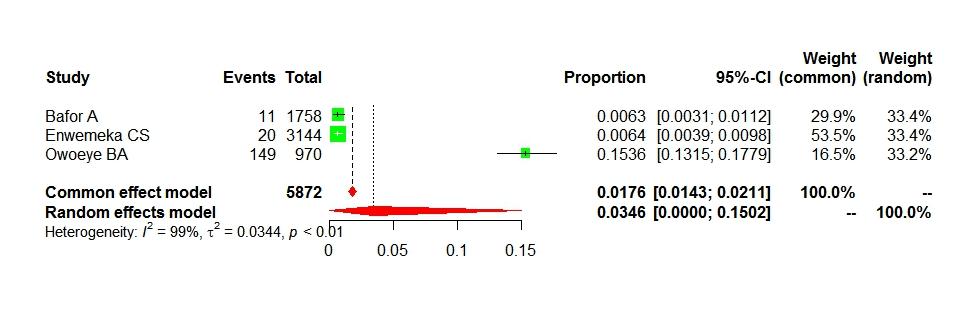

Supplement: Supplementary file 1 — Additional file 1: eFigure 1. Forest Plot of the Prevalence of HV by Afria. eFigure 2. Forest Plot of the Prevalence of HV by Asia. eFigure 3. Forest Plot of the Prevalence of HV by Europe. eFigure 4. Forest Plot of the Prevalence of HV by North America. eFigure 5. Forest Plot of the Prevalence of HV by Oceania. eFigure 6. Forest Plot of the Prevalence of HV by male. eFigure 7. Forest Plot of the Prevalence of HV by female. eFigure 8. Forest Plot of the Prevalence of HV by 0-20years. eFigure 9. Forest Plot of the Prevalence of HV by 21-60year. eFigure 10. Forest Plot of the Prevalence of HV by 61 year older. eFigure 11. Egger test. eTable 1. Quality assessment. Appendix 1. Search Strategy. [file 13047_2023_661_MOESM1_ESM.zip › 13047_661_Supplementary Materials and Appendix/eFigure 1 Prevalence of HV by Afria.jpeg]

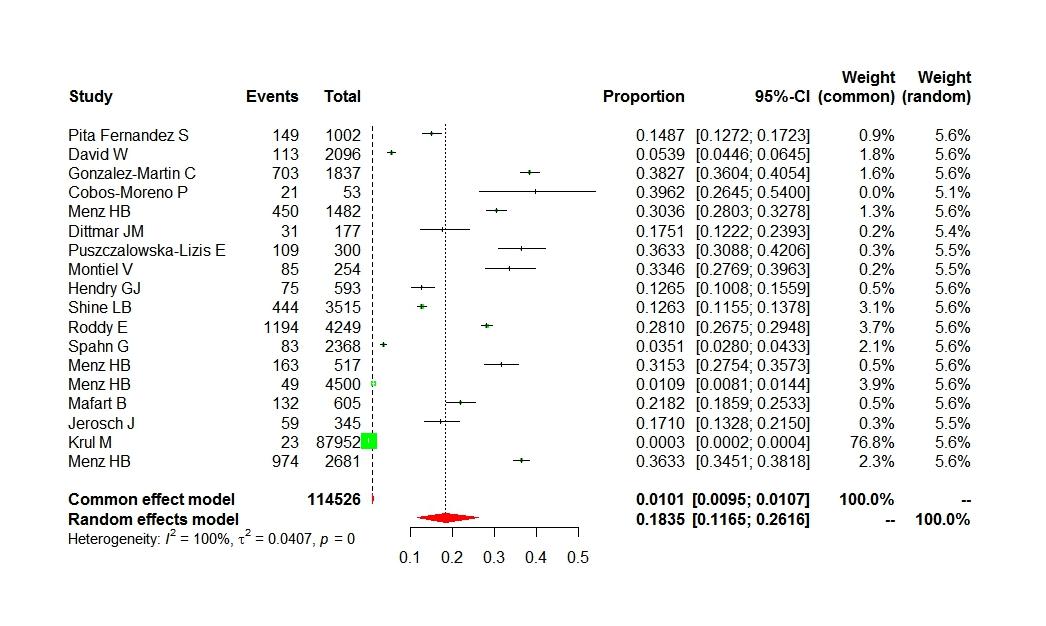

Supplement: Supplementary file 1 — Additional file 1: eFigure 1. Forest Plot of the Prevalence of HV by Afria. eFigure 2. Forest Plot of the Prevalence of HV by Asia. eFigure 3. Forest Plot of the Prevalence of HV by Europe. eFigure 4. Forest Plot of the Prevalence of HV by North America. eFigure 5. Forest Plot of the Prevalence of HV by Oceania. eFigure 6. Forest Plot of the Prevalence of HV by male. eFigure 7. Forest Plot of the Prevalence of HV by female. eFigure 8. Forest Plot of the Prevalence of HV by 0-20years. eFigure 9. Forest Plot of the Prevalence of HV by 21-60year. eFigure 10. Forest Plot of the Prevalence of HV by 61 year older. eFigure 11. Egger test. eTable 1. Quality assessment. Appendix 1. Search Strategy. [file 13047_2023_661_MOESM1_ESM.zip › 13047_661_Supplementary Materials and Appendix/eFigure 3 Prevalence of HV by Europe.jpeg]

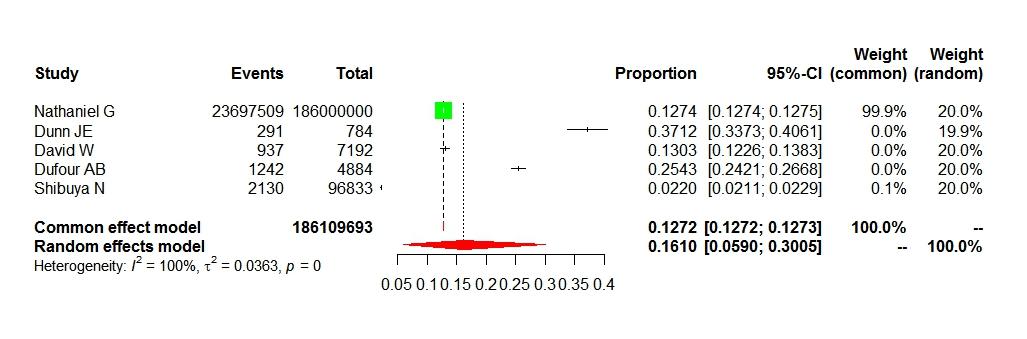

Supplement: Supplementary file 1 — Additional file 1: eFigure 1. Forest Plot of the Prevalence of HV by Afria. eFigure 2. Forest Plot of the Prevalence of HV by Asia. eFigure 3. Forest Plot of the Prevalence of HV by Europe. eFigure 4. Forest Plot of the Prevalence of HV by North America. eFigure 5. Forest Plot of the Prevalence of HV by Oceania. eFigure 6. Forest Plot of the Prevalence of HV by male. eFigure 7. Forest Plot of the Prevalence of HV by female. eFigure 8. Forest Plot of the Prevalence of HV by 0-20years. eFigure 9. Forest Plot of the Prevalence of HV by 21-60year. eFigure 10. Forest Plot of the Prevalence of HV by 61 year older. eFigure 11. Egger test. eTable 1. Quality assessment. Appendix 1. Search Strategy. [file 13047_2023_661_MOESM1_ESM.zip › 13047_661_Supplementary Materials and Appendix/eFigure 4 Prevalence of HV by North America.jpeg]

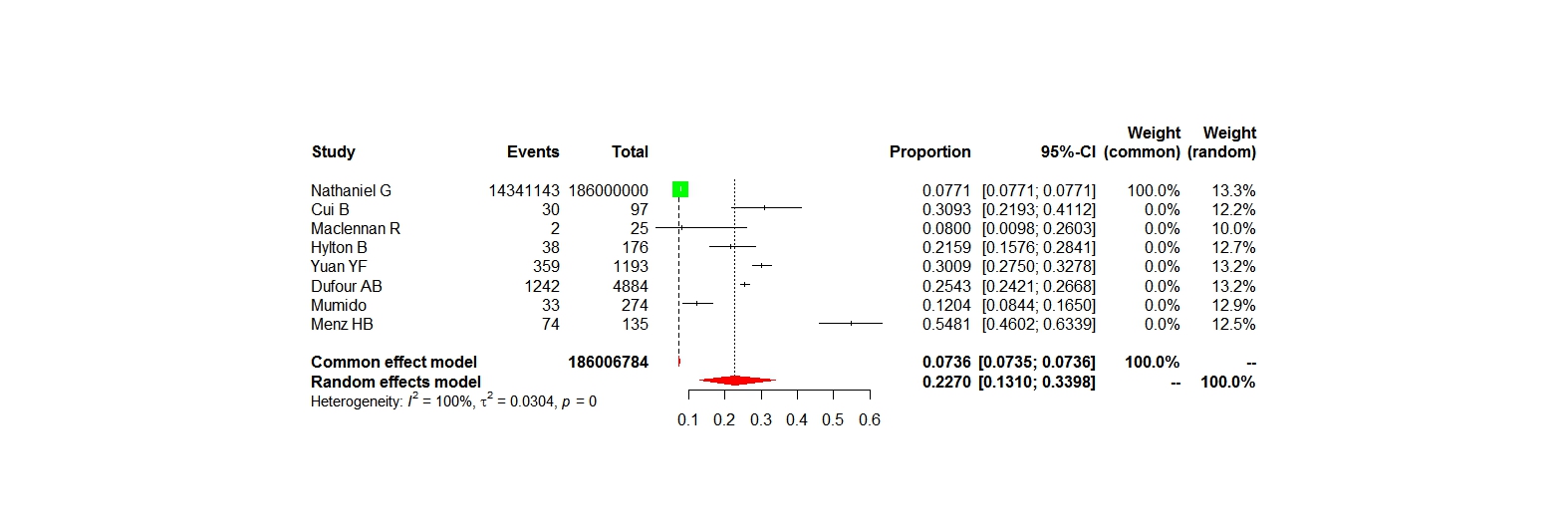

Supplement: Supplementary file 1 — Additional file 1: eFigure 1. Forest Plot of the Prevalence of HV by Afria. eFigure 2. Forest Plot of the Prevalence of HV by Asia. eFigure 3. Forest Plot of the Prevalence of HV by Europe. eFigure 4. Forest Plot of the Prevalence of HV by North America. eFigure 5. Forest Plot of the Prevalence of HV by Oceania. eFigure 6. Forest Plot of the Prevalence of HV by male. eFigure 7. Forest Plot of the Prevalence of HV by female. eFigure 8. Forest Plot of the Prevalence of HV by 0-20years. eFigure 9. Forest Plot of the Prevalence of HV by 21-60year. eFigure 10. Forest Plot of the Prevalence of HV by 61 year older. eFigure 11. Egger test. eTable 1. Quality assessment. Appendix 1. Search Strategy. [file 13047_2023_661_MOESM1_ESM.zip › 13047_661_Supplementary Materials and Appendix/eFigure10 Prevalence of HV by 61year older.jpeg]

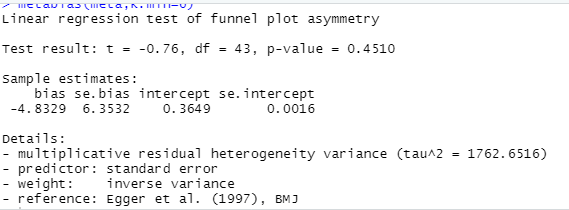

Supplement: Supplementary file 1 — Additional file 1: eFigure 1. Forest Plot of the Prevalence of HV by Afria. eFigure 2. Forest Plot of the Prevalence of HV by Asia. eFigure 3. Forest Plot of the Prevalence of HV by Europe. eFigure 4. Forest Plot of the Prevalence of HV by North America. eFigure 5. Forest Plot of the Prevalence of HV by Oceania. eFigure 6. Forest Plot of the Prevalence of HV by male. eFigure 7. Forest Plot of the Prevalence of HV by female. eFigure 8. Forest Plot of the Prevalence of HV by 0-20years. eFigure 9. Forest Plot of the Prevalence of HV by 21-60year. eFigure 10. Forest Plot of the Prevalence of HV by 61 year older. eFigure 11. Egger test. eTable 1. Quality assessment. Appendix 1. Search Strategy. [file 13047_2023_661_MOESM1_ESM.zip › 13047_661_Supplementary Materials and Appendix/eFigure11 Egger test.png]

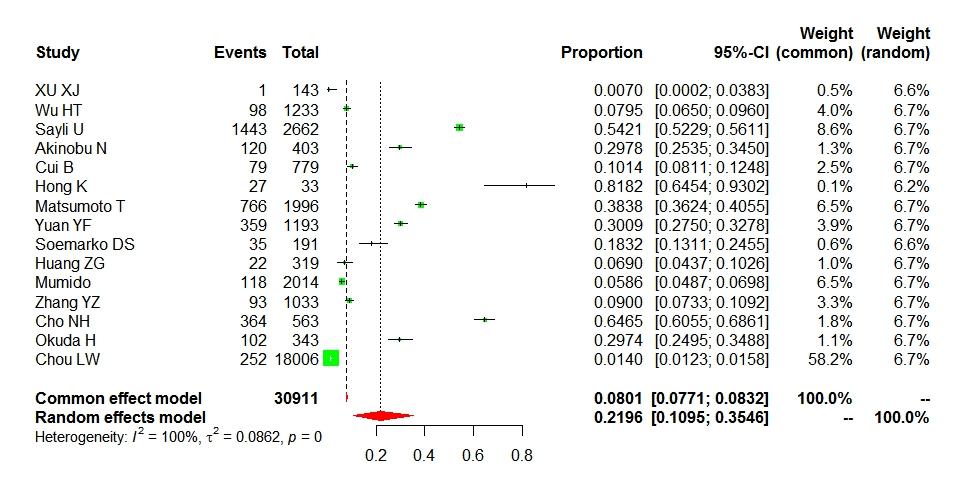

Supplement: Supplementary file 1 — Additional file 1: eFigure 1. Forest Plot of the Prevalence of HV by Afria. eFigure 2. Forest Plot of the Prevalence of HV by Asia. eFigure 3. Forest Plot of the Prevalence of HV by Europe. eFigure 4. Forest Plot of the Prevalence of HV by North America. eFigure 5. Forest Plot of the Prevalence of HV by Oceania. eFigure 6. Forest Plot of the Prevalence of HV by male. eFigure 7. Forest Plot of the Prevalence of HV by female. eFigure 8. Forest Plot of the Prevalence of HV by 0-20years. eFigure 9. Forest Plot of the Prevalence of HV by 21-60year. eFigure 10. Forest Plot of the Prevalence of HV by 61 year older. eFigure 11. Egger test. eTable 1. Quality assessment. Appendix 1. Search Strategy. [file 13047_2023_661_MOESM1_ESM.zip › 13047_661_Supplementary Materials and Appendix/eFigure2 Prevalence of HV by Asia.jpeg]

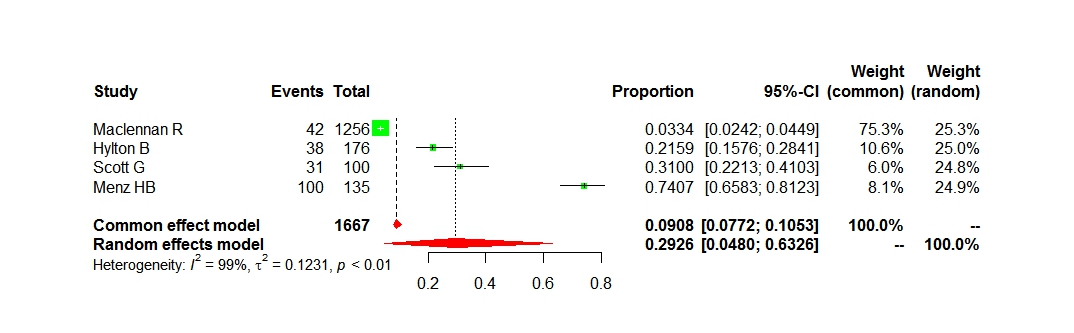

Supplement: Supplementary file 1 — Additional file 1: eFigure 1. Forest Plot of the Prevalence of HV by Afria. eFigure 2. Forest Plot of the Prevalence of HV by Asia. eFigure 3. Forest Plot of the Prevalence of HV by Europe. eFigure 4. Forest Plot of the Prevalence of HV by North America. eFigure 5. Forest Plot of the Prevalence of HV by Oceania. eFigure 6. Forest Plot of the Prevalence of HV by male. eFigure 7. Forest Plot of the Prevalence of HV by female. eFigure 8. Forest Plot of the Prevalence of HV by 0-20years. eFigure 9. Forest Plot of the Prevalence of HV by 21-60year. eFigure 10. Forest Plot of the Prevalence of HV by 61 year older. eFigure 11. Egger test. eTable 1. Quality assessment. Appendix 1. Search Strategy. [file 13047_2023_661_MOESM1_ESM.zip › 13047_661_Supplementary Materials and Appendix/eFigure5 Prevalence of HV by Oceania.jpeg]

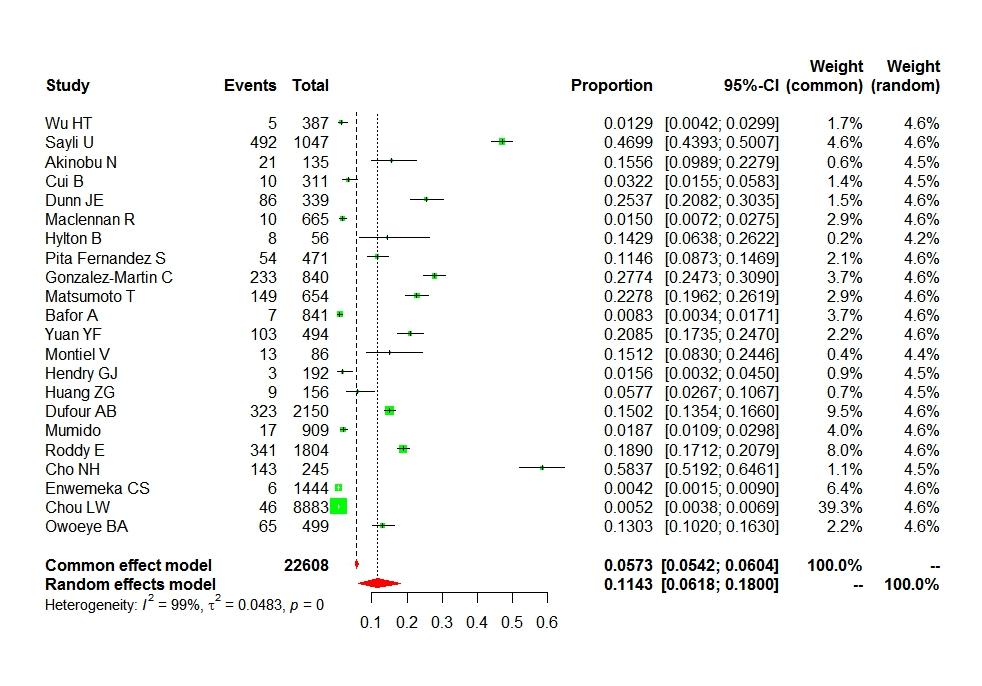

Supplement: Supplementary file 1 — Additional file 1: eFigure 1. Forest Plot of the Prevalence of HV by Afria. eFigure 2. Forest Plot of the Prevalence of HV by Asia. eFigure 3. Forest Plot of the Prevalence of HV by Europe. eFigure 4. Forest Plot of the Prevalence of HV by North America. eFigure 5. Forest Plot of the Prevalence of HV by Oceania. eFigure 6. Forest Plot of the Prevalence of HV by male. eFigure 7. Forest Plot of the Prevalence of HV by female. eFigure 8. Forest Plot of the Prevalence of HV by 0-20years. eFigure 9. Forest Plot of the Prevalence of HV by 21-60year. eFigure 10. Forest Plot of the Prevalence of HV by 61 year older. eFigure 11. Egger test. eTable 1. Quality assessment. Appendix 1. Search Strategy. [file 13047_2023_661_MOESM1_ESM.zip › 13047_661_Supplementary Materials and Appendix/efigure6 Prevalence of HV by male.jpeg]

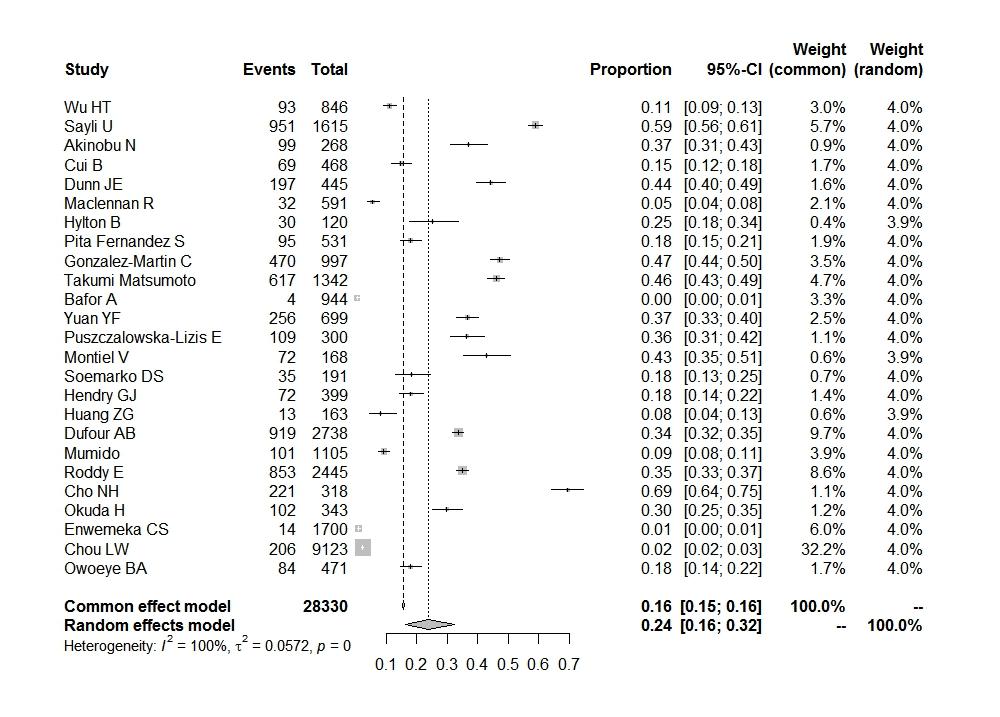

Supplement: Supplementary file 1 — Additional file 1: eFigure 1. Forest Plot of the Prevalence of HV by Afria. eFigure 2. Forest Plot of the Prevalence of HV by Asia. eFigure 3. Forest Plot of the Prevalence of HV by Europe. eFigure 4. Forest Plot of the Prevalence of HV by North America. eFigure 5. Forest Plot of the Prevalence of HV by Oceania. eFigure 6. Forest Plot of the Prevalence of HV by male. eFigure 7. Forest Plot of the Prevalence of HV by female. eFigure 8. Forest Plot of the Prevalence of HV by 0-20years. eFigure 9. Forest Plot of the Prevalence of HV by 21-60year. eFigure 10. Forest Plot of the Prevalence of HV by 61 year older. eFigure 11. Egger test. eTable 1. Quality assessment. Appendix 1. Search Strategy. [file 13047_2023_661_MOESM1_ESM.zip › 13047_661_Supplementary Materials and Appendix/efigure7 Prevalence of HV by female.jpeg]

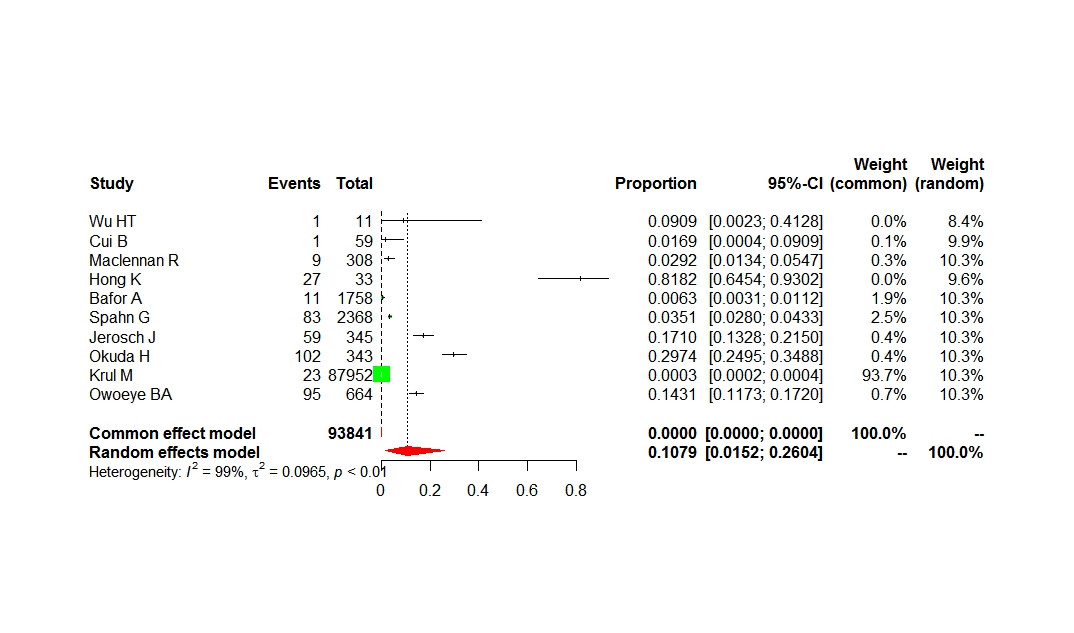

Supplement: Supplementary file 1 — Additional file 1: eFigure 1. Forest Plot of the Prevalence of HV by Afria. eFigure 2. Forest Plot of the Prevalence of HV by Asia. eFigure 3. Forest Plot of the Prevalence of HV by Europe. eFigure 4. Forest Plot of the Prevalence of HV by North America. eFigure 5. Forest Plot of the Prevalence of HV by Oceania. eFigure 6. Forest Plot of the Prevalence of HV by male. eFigure 7. Forest Plot of the Prevalence of HV by female. eFigure 8. Forest Plot of the Prevalence of HV by 0-20years. eFigure 9. Forest Plot of the Prevalence of HV by 21-60year. eFigure 10. Forest Plot of the Prevalence of HV by 61 year older. eFigure 11. Egger test. eTable 1. Quality assessment. Appendix 1. Search Strategy. [file 13047_2023_661_MOESM1_ESM.zip › 13047_661_Supplementary Materials and Appendix/eFigure8 Prevalence of HV by 0 - 20years.jpeg]

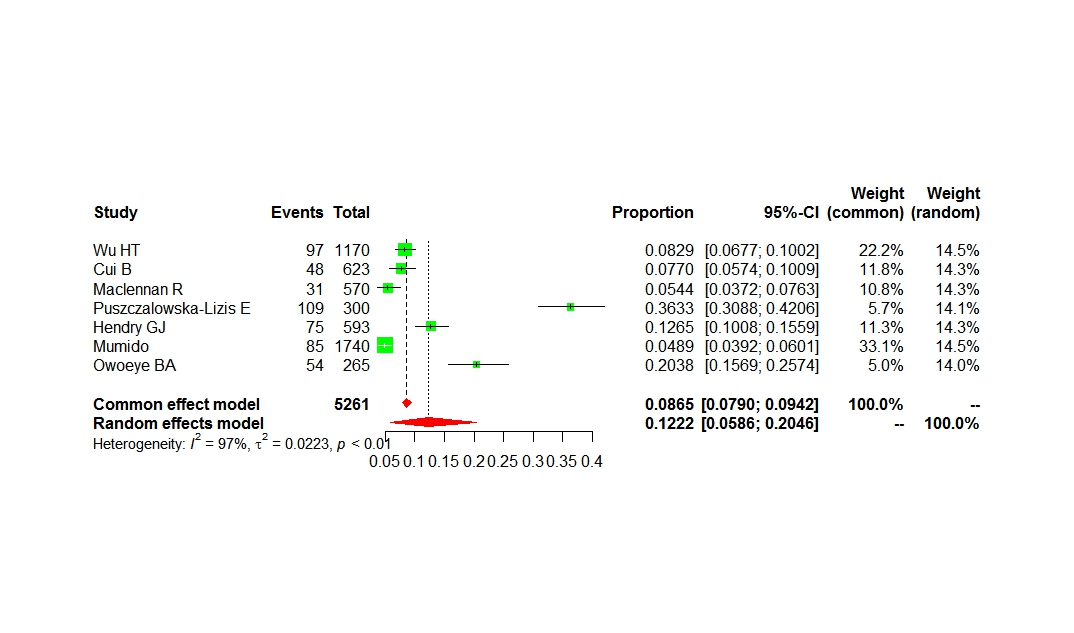

Supplement: Supplementary file 1 — Additional file 1: eFigure 1. Forest Plot of the Prevalence of HV by Afria. eFigure 2. Forest Plot of the Prevalence of HV by Asia. eFigure 3. Forest Plot of the Prevalence of HV by Europe. eFigure 4. Forest Plot of the Prevalence of HV by North America. eFigure 5. Forest Plot of the Prevalence of HV by Oceania. eFigure 6. Forest Plot of the Prevalence of HV by male. eFigure 7. Forest Plot of the Prevalence of HV by female. eFigure 8. Forest Plot of the Prevalence of HV by 0-20years. eFigure 9. Forest Plot of the Prevalence of HV by 21-60year. eFigure 10. Forest Plot of the Prevalence of HV by 61 year older. eFigure 11. Egger test. eTable 1. Quality assessment. Appendix 1. Search Strategy. [file 13047_2023_661_MOESM1_ESM.zip › 13047_661_Supplementary Materials and Appendix/eFigure9 Forest Plot of the Prevalence of HV by 21-60year.jpeg]
